# Determinants of CPAP Adherence in Hispanics with Obstructive Sleep Apnea

**DOI:** 10.1155/2014/878213

**Published:** 2014-02-05

**Authors:** Montserrat Diaz-Abad, Wissam Chatila, Matthew R. Lammi, Irene Swift, Gilbert E. D'Alonzo, Samuel L. Krachman

**Affiliations:** ^1^Division of Pulmonary and Critical Care Medicine, Sleep Disorders Center, University of Maryland School of Medicine, 685 West Baltimore Street, MSTF 800, Baltimore, MD 21201-1192, USA; ^2^Division of Pulmonary, Critical Care, and Sleep Medicine, Temple University School of Medicine, 3401 North Broad Street, Philadelphia, PA 19140, USA; ^3^Section of Pulmonary and Critical Care Medicine, Louisiana State University Health Sciences Center, 433 Bolivar Street New Orleans, LA 70112, USA

## Abstract

*Purpose*. We hypothesized that socioeconomic factors and a language barrier would impact adherence with continuous positive airway pressure (CPAP) among Hispanics with obstructive sleep apnea (OSA). *Methods*. Patients with OSA who were prescribed CPAP for at least 1 year and completed a questionnaire evaluating demographic data, socioeconomic status, and CPAP knowledge and adherence participated in the study. *Results*. Seventy-nine patients (26 males; 53 ± 11 yrs; body mass index (BMI) = 45 ± 9 kg/m^2^) with apnea-hypopnea index (AHI) 33 ± 30 events/hr completed the study. Included were 25 Hispanics, 39 African Americans, and 15 Caucasians, with no difference in age, AHI, CPAP use, or BMI between the groups. While there was a difference in educational level (*P* = 0.006), income level (*P* < 0.001), and employment status (*P* = 0.03) between the groups, these did not influence CPAP adherence. Instead, overall improvement in quality of life and health status and perceived benefit from CPAP influenced adherence, both for the group as a whole (*P* = 0.03, *P* = 0.004, and *P* = 0.001, resp.), as well as in Hispanics (*P* = 0.02, *P* = 0.02, *P* = 0.03, resp.). *Conclusion*. In Hispanic patients with OSA, perceived benefit with therapy, rather than socioeconomic status or a language barrier, appears to be the most important factor in determining CPAP adherence.

## 1. Introduction

Obstructive sleep apnea (OSA) syndrome is a common disorder with a reported prevalence of 2% of women and 4% of men between the ages of 30 and 60 years old [1]. Effective treatment is important due to the identification of OSA as an independent risk factor for cardiovascular disease [2–4]. While continuous positive airway pressure (CPAP) therapy is effective at correcting sleep disordered breathing [5, 6] and improving daytime sleepiness [6, 7], adherence is poor [8–10]. Although a number of factors are suggested to influence adherence, the importance of many of the determinants remains unclear [11, 12].

CPAP adherence among minorities in the United States is reported to be lower, especially among African Americans [13–15]. Factors such as the neighborhood of residence and associated socioeconomic status appear important [16–18] and are known to influence overall medical care and follow-up among minority populations [19–23]. While CPAP adherence has been examined in Caucasian and African American patients, no prior study has examined CPAP adherence in Hispanic patients with OSA. In addition to socioeconomic factors, a language barrier may play a role. We hypothesized that among Hispanics with OSA CPAP adherence would be influenced by socioeconomic factors and a language-related lack of understanding of how and why to use CPAP therapy.

## 2. Methods

### 2.1. Patient Selection

We recruited consecutive patients over a one-year period that had been previously studied in a University Hospital Sleep Laboratory. Included were patients referred from the University Sleep Clinic as well as patients referred from outside specialists with an interest in sleep medicine. Patients who were asked to participate had been newly diagnosed with OSA by an overnight polysomnogram study, had undergone a CPAP titration, and had not used CPAP before. OSA was defined as an apnea-hypopnea index (AHI) of ≥5 events/hr with symptoms of excessive daytime sleepiness or an AHI ≥15 events/hr. Patients reviewed the results of their sleep studies and discussed therapy with the referring physician, who then prescribed the patient's CPAP. Specific instructions about use and care of their CPAP device were given by the durable medical equipment company who delivered and set up the CPAP at home. Patients referred from the University Sleep Clinic and from the outside specialists are routinely seen in follow-up 6–8 weeks after CPAP initiation to evaluate adherence. Patients are then seen as often as needed to optimize adherence or to change therapy if they are unable to tolerate CPAP. Once the patients are considered stable, they are instructed to follow-up on a yearly basis. Patients who had been prescribed CPAP and had it set up at home for at least 1 year (12–15 months) prior to enrollment into the study were selected. Patients were excluded from participating in the study if they (1) had associated obesity-related alveolar hypoventilation or central sleep apnea, (2) had other conditions that might interfere with sleep (underlying chronic respiratory disorders, uncontrolled allergies, heart failure, narcolepsy, and periodic leg movements), (3) had known pregnancy, (4) were unable to be located or refused to complete or return the questionnaire, and (5) refused or were unable to sign informed consent. The study was approved by the Institutional Review Board for Human Research (Temple University School of Medicine, Philadelphia, PA, USA).

### 2.2. Questionnaire

All study materials including the questionnaire and informed consent were mailed both in English and in Spanish to each patient at their last known address. The patients were instructed to mail back both the completed questionnaire and the signed informed consent in a stamped envelope that was provided. If the patients had not returned the questionnaire after 2 weeks, they were called once using their last known telephone phone number as a reminder and asked to complete and return the questionnaire. The questionnaire consisted of 30 questions divided into 2 main sections. The first section contained questions related to demographic and socioeconomic data, including age, gender, racial/ethnic background, level of education, yearly income, country of birth, and language that was spoken. The second section contained questions related to the diagnosis of OSA and CPAP adherence. Included were questions related to symptoms, familiarity with CPAP therapy, perceived benefit from therapy, and CPAP adherence. Good adherence was defined as reported use of CPAP at least 4 hours per night, 70% of the nights of the week [8]. The section also included questions related to the level of support from medical and technical personnel related to the implementation of CPAP.

### 2.3. Polysomnogram

Polysomnograms were performed in-laboratory and consisted of a recording of rib cage and abdominal motion (Respitrace; Ambulatory Monitoring; White Plains, NY, USA), with air flow measured using a pressure transducer. Snoring was monitored using a snore microphone. Other recordings included pulse oximetry (Cephalo Pro, Viasys Healthcare, Yorba Linda, CA, USA), electrocardiogram, electrooculogram, digastric electromyogram, and electroencephalogram. All variables were continuously recorded and stored in a computerized system (Viasys Healthcare; Yorba Linda, CA, USA). Sleep was staged and arousal defined using established criteria [24]. Obstructive apnea was defined by the lack of airflow for ≥10 seconds, associated with the presence of rib cage and abdominal movement [24]. Obstructive hypopnea was defined by a 50% decrease in airflow associated with a ≥3% decrease in oxygen saturation or an arousal [24]. Apnea was defined as central if there was a lack of respiratory effort during the period of absent airflow [24]. The AHI was calculated as the number of apneic and hypopneic events per hour of sleep. An arousal was defined as an abrupt shift of EEG frequency including alpha, theta, and/or frequencies >16 Hz (but not spindles) that lasts at least 3 seconds, with at least 10 seconds of stable sleep preceding the change [24]. Other calculated variables included total sleep time, sleep efficiency (total sleep time divided by time in bed), arousal index, and the percent of total sleep time with an arterial oxygen saturation (SpO_2_) <90%. All patients had a baseline polysomnogram followed by a full night CPAP titration study or had a split night study with the initial portion performed off CPAP and the remainder of the night consisting of a CPAP titration. CPAP titrations were performed using recommended guidelines [25] with the patient's prescribed CPAP pressures based on studies that were considered optimal, good, or adequate as described in the guidelines. All of the polysomnogram studies were initially scored by a single senior technologist. The same author (SK) reviewed each study.

### 2.4. Statistical Analysis

Continuous data are presented as the mean ± SD. Differences in continuous variables between the CPAP adherent and nonadherent groups were evaluated using unpaired *t*-tests. Categorical data are presented as frequencies with percentages; between-group differences were analyzed using Fisher's exact test. Differences between ethnic groups were analyzed using one-way ANOVA with follow-up pairwise comparisons using a Bonferroni adjustment. A *P* value ≤ 0.05 was considered statistically significant. All data were analyzed using SAS V9.2 software.

## 3. Results

### 3.1. Patient Characteristics

A total of 219 patients who were diagnosed with OSA and treated with CPAP therapy over a one-year period were selected to participate in the study. Of the 219 questionnaires that were mailed, 79 patients (26 males, 53 ± 11 yrs, and BMI = 45 ± 9 kg/m^2^) responded (36%) and returned their questionnaires (Table 1) and included 25 Hispanics, 39 African Americans, and 15 Caucasians (Table 1). Twenty questionnaires (9%) were returned unopened (incorrect address). The respondents had evidence of severe OSA with an AHI of 33 ± 30 events/hr with a mean CPAP requirement of 12 ± 4 cm H_2_O (Table 1). The three population groups were similar in regard to age, sex, BMI, AHI, sleep quality, and CPAP requirement (Table 1). There was no difference in AHI or racial/ethnic distribution between the responders and nonresponders (*P* = 0.5 and *P* = 0.3, resp.).

### 3.2. CPAP Adherence

Overall, 63 patients reported that they are still using CPAP therapy, an average of 5.9 ± 1.9 hours/night and 5.7 ± 1.7 nights/week. There was no difference in the AHI in the group that was adherent with CPAP (35 ± 30 events/hr) compared to the group that was not using CPAP (24 ± 21 events/hr) (*P* = 0.12). However, the prescribed CPAP pressure was higher in the adherent group (12.9 ± 3.5 cm H_2_O) compared to the nonadherent group (10.2 ± 2.8 H_2_O) (*P* = 0.01). CPAP adherence was similar between the groups, both in regard to the number of nights/week (Caucasian = 5.5 ± 1.4; African American = 5.4 ± 2.0; Hispanics = 5.6 ± 1.8; *P* = 0.9) and hours/night (Caucasian = 6.4 ± 1.5; African Americans = 5.5 ± 1.8; Hispanics = 5.8 ± 2.2; *P* = 0.8) CPAP was used. The percentage of patients considered to have good adherence to CPAP was also similar between the groups: 46.4% of Caucasians, 56.4% of African Americans, and 48% of Hispanics; *P* = 0.73.

### 3.3. Socioeconomic and Language Differences

**Figure 1:**
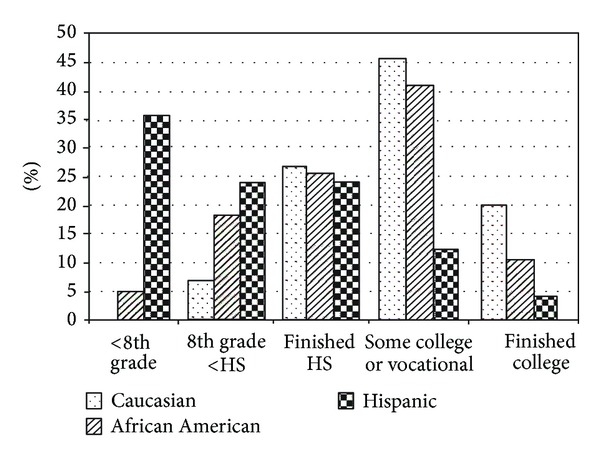
There was a significant difference in educational level between the 3 racial/ethnic groups (*P* = 0.006). Group by group comparison: Caucasian versus African American: *P* = 0.7; Caucasian versus Hispanic: *P* = 0.04; African American versus Hispanic: *P* = 0.01. HS = High school.

**Figure 2:**
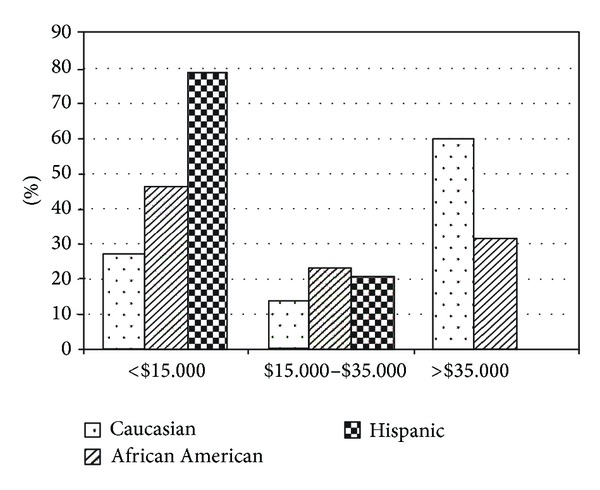
Between the 3 racial/ethnic groups there was a significant difference in income level (*P* < 0.001). Group by group comparison: Caucasian versus African American: *P* = 0.2; Caucasian versus Hispanic: *P* < 0.001; African American versus Hispanic: *P* = 0.003.

**Figure 3:**
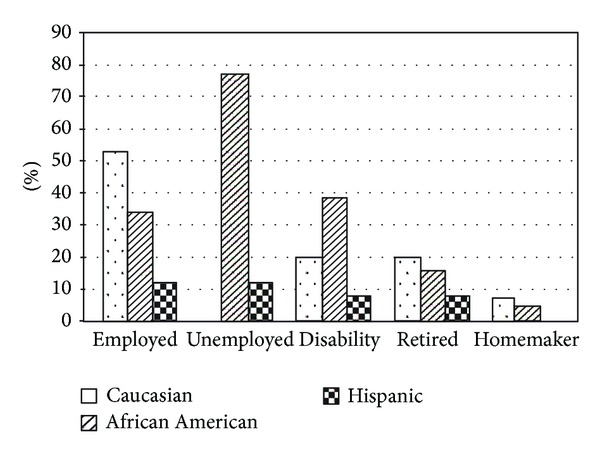
In regard to employment status, there was a significant difference between the 3 racial/ethnic groups (*P* = 0.03). Group by group comparison: Caucasian versus African American: *P* = 0.47; Caucasian versus Hispanic: *P* = 0.02; African American versus Hispanic: *P* = 0.1.

Between the 3 racial/ethnic groups, there was a difference in the educational level (*P* = 0.006), with 36% of the Hispanics having less than an 8th grade education versus none in the Caucasian group and 5% in the African American group (Figure 1). In addition, there was a difference between groups in regard to income level (*P* < 0.001), with 79% of Hispanics earning less than $15,000/yr versus 46% of African Americans and 27% of Caucasians (Figure 2). In relation to employment status, the Caucasian group was more often employed (Caucasian = 53%, African American = 33%, and Hispanics = 12%; *P* = 0.03) (Figure 3). In the Hispanic group, 80% were born outside the continental United States, having lived in the United States 29 ± 13 yrs, and 33% of Hispanics spoke only Spanish. Spoken language did not influence adherence, both in the combined patient group (*P* = 0.35) and within the Hispanic group (*P* = 0.64). Fifty-two percent of Hispanics reported that their physician made sure that the results of their sleep studies were explained in their language, 71% reported that the home company that set up their equipment had someone who spoke their language, and 91% stated that the home company adequately explained how to use their CPAP. However, on analysis, none of these variables appeared to influence CPAP adherence.

### 3.4. Determinants of CPAP Adherence

In regard to the determinants that were associated with CPAP adherence in the group as a whole, compared to the patients that were no longer using CPAP, those using CPAP more often reported an overall improvement in quality of life (*P* = 0.03) and health status (*P* = 0.004). In addition, those adherent with CPAP reported a greater perceived benefit from using CPAP (*P* = 0.001). Similar to the group as a whole, Hispanics who were adherent with CPAP more often noted an overall improvement in quality of life (*P* = 0.02) and health status (*P* = 0.02), compared to those that were no longer using CPAP. In addition, Hispanics who were adherent with CPAP therapy more often reported an initial improvement in symptoms (*P* = 0.003), as well as a greater perceived benefit from using CPAP (*P* = 0.03).

## 4. Discussion

While CPAP is an effective therapy in the treatment of OSA, the determinants that influence CPAP adherence are not completely defined [11, 12]. The influence that socioeconomic factors and a language barrier may have on CPAP adherence in Hispanic patients has not been fully investigated. There are 2 main findings in this study: (1) CPAP adherence in Hispanics is not related to noted differences in socioeconomic status or spoken language and (2) the main factors associated with CPAP adherence in Hispanics are related to perceived benefit and improvement in quality of life and health status noted with treatment.

We had hypothesized that CPAP adherence would be influenced in Hispanics by socioeconomic status and a language barrier. However, these did not turn out to be determinants in regard to CPAP adherence. In the present study, for Hispanic patients and for the group as whole, CPAP adherence was associated with an overall improvement in quality of life and health status and a greater perceived benefit from using CPAP. In addition, Hispanic patients adherent with CPAP reported an initial improvement in their symptoms with the use of CPAP therapy.

Similar results have been reported by others. Kribbs et al. objectively examined CPAP usage in 35 patients with OSA [8]. At the initial 1-month follow-up, the 16 patients considered to be regular CPAP users (≥4 hrs/night on ≥70% of the days) reported more satisfaction with CPAP treatment and had a better level of energy during the day compared to the 19 patients who were irregular users of CPAP [8]. More recently, Wells et al. prospectively examined CPAP adherence at 30 days in 54 patients with OSA [26], noting that CPAP adherence was associated with an improvement in OSA symptoms.

In healthcare, racial differences have been reported when it comes to medication adherence [19, 20], with a known effect on diabetes and blood pressure control in minority populations [20–23]. In the treatment of OSA, racial differences in CPAP adherence have been noted by some [14, 15] but not all investigators [27]. Billings et al. examined the racial differences in CPAP adherence in 191 patients who were part of the HomePAP study [14]. At both 1 and 3 months, race was a predictor of CPAP use, with African American patients being less adherent based on the number of minutes of use per night as well as the percent of days that CPAP was used for ≥ 4 hrs/night. Means et al. found similar results in 499 veterans whose CPAP adherence data was reviewed from a clinical database at 30 and 90 days [15]. CPAP adherence was significantly higher in Caucasians versus African Americans at both time points based on the percentage of nights used as well the average hours per night. The presence of a mental health diagnosis had a negative impact on CPAP adherence in the African American patients but not in the Caucasian group [15].

Budhiraja et al. noted a lower daily CPAP usage in African Americans compared to Caucasians in 100 patients who were being studied to evaluate if CPAP adherence in the first few days of use predicted adherence at 30 days [13]. The study was limited by the lack of educational and socioeconomic data in assessing racial differences in adherence. Joo and Herdegen noted that African Americans were 5 times more likely to be nonadherent with CPAP therapy at 30 days as compared to Caucasians [18]. However, the results may have been biased by the much smaller number of Caucasians versus African Americans in the study. All of these studies are limited by the fact that they evaluated adherence for not more than 90 days and none of them evaluated adherence in Hispanic patients.

In contrast to the above studies, Scharf et al. evaluated self-reported CPAP adherence in 108 patients 3–9 months after they were diagnosed and treated with CPAP therapy [27]. There was no difference between African Americans and Caucasians both in regard to initial acceptance of CPAP therapy (48% versus 45%, resp.) and more long-term adherence (39% versus 35%, resp.). Age, Epworth Score, and respiratory disturbance index (RDI) were found to be associated with long-term CPAP adherence, but race was not. In the present study, CPAP adherence in Hispanics was similar to the other groups. In addition, unlike previous studies, we evaluated adherence after the patient had been prescribed CPAP for at least 1 year.

Socioeconomic factors have been suggested to influence CPAP adherence [14, 16, 17]. Simon-Tuval et al. evaluated CPAP acceptance in 162 patients with OSA who completed a 2-week adaptation period [17]. CPAP acceptance, as measured 4–6 weeks following completion of the adaptation period, was significantly greater in patients from a higher income level. Platt et al. retrospectively evaluated the importance of neighborhood-level socioeconomic status on CPAP adherence in 266 veterans with OSA [16]. After 1 week of use, CPAP adherence was found to be lower in patients from a low socioeconomic neighborhood (34%) compared to patients from a high socioeconomic neighborhood (62%). However, Billings et al. found that socioeconomic status by itself had no influence on CPAP adherence when evaluated at both 1 and 3 months [14]. In the present study, we also did not find socioeconomic status to influence CPAP adherence, despite significant differences in yearly income and educational levels, with 79% of Hispanics earning less than $15,000/yr and 36% having less than an 8th grade education.

There are a number of limitations within the study that need to be addressed. First, the study involved a relatively small number of patients. However, the number of patients evaluated is similar or higher than that of previous studies that have examined determinants of CPAP adherence [18, 26]. Second, CPAP adherence was self-assessed with the use of a questionnaire that was mailed to the patients, rather than being quantitatively monitored. Subjective assessment has been shown to overestimate actual CPAP usage by approximately 1 hour per night [28]. However, this would have affected all groups proportionally and be less likely to affect the main objective of the study, the evaluation of factors associated with CPAP adherence in Hispanics. Third, this was a cross-sectional survey of patients prescribed CPAP for at least 1 year and did not evaluate factors associated with early CPAP adherence. Finally, the results may not be representative of all Hispanics. Our urban Hispanic population consists mostly of immigrants from Puerto Rico. Whether similar findings apply to Hispanics in other demographic areas will have to be determined.

In conclusion, in Hispanics with OSA, there is no evidence that a language barrier is important in determining CPAP adherence. In addition, while the socioeconomic status was substantially lower in Hispanics patients, the perceived benefit and improvement in quality of life and health status obtained with CPAP therapy were the most important determinants for CPAP adherence. Larger, prospective studies are needed to confirm these findings.

## Figures and Tables

**Table 1:** Baseline characteristics*.

Variable	Total group	Hispanic	African American	Caucasian	*P* value
Patients, *n*	79	25	39	15	
Age, yrs	53 ± 11	54 ± 12	54 ± 13	56 ± 12	*P* = 0.8
Male, *n* (%)	26 (33)	8 (32)	7 (18)	3 (20)	*P* = 0.4
BMI, kg/m^2^	45 ± 9	44 ± 7	40 ± 9	46 ± 13	*P* = 0.7
Baseline AHI, events/hr	33 ± 30	32 ± 24	34 ± 31	32 ± 38	*P* = 0.3
Lowest SpO_2_, %	82 ± 9	85 ± 5	81 ± 10	81 ± 7	*P* = 0.3
% TST SpO_2_ < 90%	8 ± 13	11 ± 16	8 ± 12	5 ± 7	*P* = 0.7
TST, min	308 ± 77	330 ± 70	324 ± 76	279 ± 79	*P* = 0.2
Sleep efficiency, %	78 ± 17	82 ± 15	80 ± 15	74 ± 19	*P* = 0.4
Arousal index, events/hr	18 ± 14	17 ± 10	17 ± 15	24 ± 16	*P* = 0.2
Sleep architecture, %TST					
N1, %	16 ± 14	16 ± 14	15 ± 12	17 ± 18	*P* = 0.9
N2, %	53 ± 16	51 ± 19	53 ± 13	56 ± 13	*P* = 0.7
N3, %	13 ± 11	12 ± 10	14 ± 11	13 ± 10	*P* = 0.8
REM, %	16 ± 13	16 ± 15	17 ± 9	14 ± 12	*P* = 0.7
CPAP, cm H_2_O	12 ± 4	12 ± 3	13 ± 4	12 ± 4	*P* = 0.7

*Data are presented as mean ± SD or the number of patients. BMI: body mass index; AHI: apnea-hypopnea index; TST: total sleep time; REM: rapid eye movement; CPAP: continuous positive airway pressure.
